# Memory effects can make the transmission capability of a communication channel uncomputable

**DOI:** 10.1038/s41467-018-03428-0

**Published:** 2018-03-20

**Authors:** David Elkouss, David Pérez-García

**Affiliations:** 1 QuTech, Delft University of Technology, Lorentzweg 1, 2628 CJ Delft, The Netherlands; 2 Departamento de Análisis Matemático and Instituto de Matemática Interdisciplinar, Universidad Complutense de Madrid, 28040 Madrid, Spain; 3 ICMAT, c/ Nicolás Cabrera, Campus de Cantoblanco, 28049 Madrid, Spain

## Abstract

Most communication channels are subjected to noise. One of the goals of information theory is to add redundancy in the transmission of information so that the information is transmitted reliably and the amount of information transmitted through the channel is as large as possible. The maximum rate at which reliable transmission is possible is called the capacity. If the channel does not keep memory of its past, the capacity is given by a simple optimization problem and can be efficiently computed. The situation of channels with memory is less clear. Here we show that for channels with memory the capacity cannot be computed to within precision 1/5. Our result holds even if we consider one of the simplest families of such channels—information-stable finite state machine channels—restrict the input and output of the channel to 4 and 1 bit respectively and allow 6 bits of memory.

## Abstract (web summary)

In information theory one is interested in how much information can be reliably sent over noisy communication channels. Here the authors show that for channels with memory the optimal rate of information transmission is uncomputable.

## Introduction

The need to manipulate large amounts of information is one of the main characteristics of our society. It is crucial to protect the information against noise and errors in order to ensure its reliable transmission and long-term storage. It is important also to do so in the optimal way so that communication channels transmit and memories store trustworthily as much information as possible. This problem motivated Shannon^1^, already in 1948, to develop the theory of communications. The natural problem that Shannon posed is, given a noisy communication channel, find the maximum rate of information it can transmit with an arbitrarily small error.

In an ingenuity tour de force, he proved that for channels that keep no memory of their past uses (called memoryless), this quantity—the capacity of the channel—defined in such operational way, has a simple entropic expression. It coincides with the maximization, on all inputs to the channel, of the so-called mutual information between input and output in one single use of the channel. This coding result was complemented years later by the Blahut-Arimoto (BA) algorithm^2,3^, which allows to efficiently approximate the capacity of any memoryless channel within any desired precision.

The situation for channels with memory is less clear. Regarding coding theorems, more and more general classes of channels were successfully dealt with^4–7^ culminating in the generalized capacity formula^8^. In this last work, Verdu and Han derived a generalization of Shannon’s coding theorem which essentially makes no assumption regarding the structure of the channel. When it comes to algorithms that approximate the capacity, despite considerable effort, the situation is nowadays less successful. Even if we restrict to the simplest case of channels with memory, the so-called finite state machine channels (FSMCs), the problem remains open. There is a rich literature dealing with particular cases (see e.g. refs. ^9–19^). However, these results do not address FSMCs in full generality or cannot guarantee the precision of the result.

It is the main aim of this work to show that an algorithm that computes approximately the capacity of an arbitrary FSMC cannot exist. Since computable functions are exactly those that can be computed by an algorithm, this is equivalent to show that any function that approximates sufficiently the capacity of any FSMC must necessarily be uncomputable.

## Results

### Main statements

Aiming at an impossibility result, the simpler the family of channels we consider, the stronger the result. This is why we consider FSMCs. The same result hence holds true for any more general family of channels with memory.

In order to be precise, an FSMC with *n* possible input symbols (the number of possible output symbols will be always 2) and *m* possible states in the memory is determined by^9^ a set of conditional probability assignments. The set of conditional probability assignments $p(y,s|x,s^\prime )$ describes the probability of output symbol *y* and transition to state *s* in the memory if the FSMC is in state *s*′ and gets *x* as input. Moreover, we will only consider FSMCs in which the initial state is fixed and known to the sender and receiver. We denote the initial state by *s*_0_.

To avoid problems of approximating *p*(*y*, *s*|*x*, *s*′) we will only consider FSMCs for which the probability assignments *p*(*y*, *s*|*x*, *s*′) are rational numbers. Moreover, we will only consider FSMCs for which *p*(*y*, *s*|*x*, *s*′) are in product form *p*(*y*|*x*, *s*′) *p*(*s*|*x*, *s*′) and which are information stable. Information stable channels are one of the simplest classes of channels with memory. For these channels the capacity is given by the limit of the mutual information rate^20^ and it is not necessary to consider the most general capacity formula^8^.

Our main result can then be stated as

*Main Result 1*: Any function that on input the set of probability assignments {*p*(*y*|*x*, *s*′), *p*(*s*|*x*, *s*′)}_*s*, *y*, *x*, *s*′_ of an information stable FSMC **N** with 10 input symbols and 62 states, outputs a rational number* c *so that the capacity of **N** verifies

1 $$\left| {C({\mathbf{N}}) - c} \right| \le \frac{1}{5},$$

must be uncomputable.

It is obvious that the same result then holds for *n* input symbols and *m* states as long as *n* ≥ 10 and *m* ≥ 62. For example, taking *n* = 16 and *m* = 64 we get a channel with 4 bits of input, 1 bit of output and 6 bits of memory.

Indeed, we will prove something slightly stronger. Let us recall that a decision problem can be cast as a function with values in {0, 1}, where 1 stands for accept and 0 for reject. When the associated function is uncomputable, the decision problem is called undecidable.

Fix a rational number *λ* ∈ (0, 1]. We will give explicitly a subfamily ${\cal S}_\lambda$ of FSMCs (information stable and with rational conditional probability assignments in product form) with 10 input symbols and 62 states, with the additional property that all channels ${\mathbf{N}} \in {\cal S}_\lambda$ have capacity ≥*λ* or ≤*λ*/2.

*Main Result 2:* It is undecidable to know whether ${\mathbf{N}} \in {\cal S}_\lambda$, given by its set of probability assignments {*p*(*y*|*x*, *s*′), *p*(*s*|*x*, *s*′)}_*s*, *y*, *x*, *s*′_, has capacity ≥λ or ≤ *λ*/2.

It is clear that if we consider our Main Result 2 for ${\cal S}_1$ we get Main Result 1. That is, if we could approximate the capacity within error 1/5 then, given a channel from ${\cal S}_1$ for which we know its capacity is ≤1/2 or ≥1, we could decide which is the case. However, we know by Main Result 2 that the problem is undecidable.

### Proof sketch

The idea behind our proof is to construct a family of channels such that the capacity of a channel in the family is related to some property of a probabilistic finite automaton (PFA). Our construction is indirect, we first give a map form PFAs to FSMCs; then we define the channel family as the set of FSMCs that are the image of a PFA via this map. The important property of this map, proved in Theorem 1, is that the capacity of a channel in the image set is given by the value of its preimage PFA (see the PFA section). We now sketch the structure of the proof and point to the appropriate sections for further detail.

**Fig. 1:**
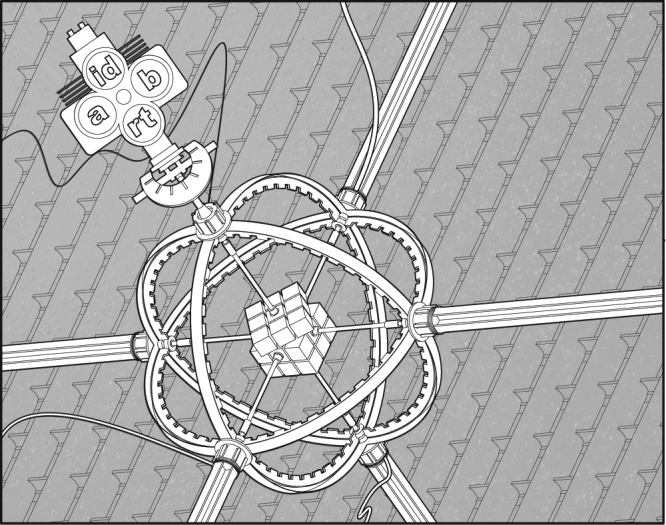
A noisy Rubik cube solver as an example of a probabilistic finite automaton (PFA). This PFA has as many states as different Rubik cube configurations. It begins in some predefined state and can be manipulated with four different buttons or input alphabet symbols: {*a*, *b*, id, rt}. A Rubik cube can be solved by combinations of only two sequences of rotations^36^. The press of the buttons *a*, *b* will, with some probability, implement one of these two sequences and with the complementary probability apply a random rotation. The buttons id, rt will make the state of the Rubik cube either stay idle or bring it back to the initial state. The accepting state is the solved configuration of the cube. The value of this automaton would be the maximum probability of taking the initial configuration to the solved configuration by pressing a sequence of buttons (Credit: Francisco García Moro)

FSMCs, defined in Supplementary Note 2, are controlled by a finite state machine. The state of the finite state machine determines the (memoryless) channel that is applied to the input. Then depending both on the input and the current state it transitions probabilistically to the next state. A PFA is a finite state machine that transitions probabilistically from state to state depending on the current state and the input (see Fig. 1). Hence, it is possible to identify the finite state machine controlling an FSMC with a PFA.

A concrete input into a PFA, that is a sequence of input symbols, is accepted if after reading the input the PFA ends in a subset of the states called accepting states, otherwise the input is rejected. Informally, the value of a PFA is the maximum probability of ending in an accepting state. It turns out that many decision problems related to the value cannot be solved. Notably, given some value *λ* ∈ (0, 1) and a PFA ${\cal A}$ it is undecidable to know if the value of ${\cal A}$ is greater than *λ*^21–23^. Here, we use a recent proof of this result by Hirvensalo^24^, see Theorem 1 in Supplementary Note 6B. In order to prove a result about approximations, we amplify this result about decision problems with a very original PFA construction by Gimbert and Oualhadj^25^, which is the key ingredient in our proof, see Lemma 2 in Supplementary Note 6A. With this construction, it is possible to embed any PFA ${\cal A}$ into a larger PFA ${\cal B}_\lambda$ (with *λ* ∈ [0, 1/2]) such that: the value of ${\cal B}_\lambda$ is ≤*λ* if and only if the value of ${\cal A}$ is ≤1/2 and the value of ${\cal B}_\lambda$ is 2*λ* if and only if the value of ${\cal A}$ is >1/2. Joining both arguments, we conclude that the value of a PFA cannot be approximated with arbitrary precision, since it is undecidable to know whether the value of a PFA is smaller than *λ* or equal to 2*λ*.

To go from there to Main Result 2 it is enough to construct for any PFA ${\cal A}$ a channel ${\mathbf V}_{\cal A}$ so that the capacity of ${\mathbf{V}}_{\cal A}$ equals the value of ${\cal A}$. The idea for that is very natural:

Consider a channel with two input registers. The first one is used to control the PFA. The second one corresponds to the data to be transmitted. If the PFA is in an accepting state the channel outputs the contents of the second input register, that is, it behaves as a noiseless channel. Otherwise it is only noise, i.e. it outputs uniformly at random a symbol from the output alphabet.

Intuitively, this map should already have the property that the capacity of ${\mathbf{V}}_{\cal A}$ equals the value of ${\cal A}$. However, without an additional gadget, we cannot conclude this. Let us see with an example why it does not suffice. Consider for instance a PFA that transitions from the initial state to an accepting state with probability 1/2 and with probability 1/2 to some other state. Moreover, suppose that these two states are final in the sense that the PFA cannot leave them once reached. Such a PFA would have value 1/2. However, the capacity of the associated channel would be zero because the error probability of any code would always be greater than 1/4. In order to solve this problem we concatenate the map with a function $\gamma ( \cdot )$ from PFAs to PFAs that adds to the PFA a reset and a freeze symbols. The reset symbol takes the PFA back to the initial state while the freeze symbol keeps the state of the PFA unchanged. We prove in Lemma 3 in Supplementary Note 6C that the value of an automaton ${\cal A}$ does not change under this map, i.e. the value of ${\cal A}$ equals the value of $\gamma ({\cal A})$. But, for PFAs with the additional reset and freeze symbols we can show the desired result that the capacity of the channel ${\mathbf{V}}_{\gamma ({\cal A})}$ equals the value of the automaton ${\cal A}$. This is our main technical result, proved in Theorem 1.

The intuition between the equality of the capacity of channel ${\mathbf{V}}_{\cal A}$ and the value of ${\cal A}$, $\mathrm{val}_{\cal A}$, is as follows. For any *δ* > 0 there exists a word with value greater than $\mathrm{val}_{\cal A} - \delta$. By feeding this word into the control register, the channel will transition into a final state with probability at least $\mathrm{val}_{\cal A} - \delta$. The state of the channel can then be frozen making the mutual information rate tend to $\mathrm{val}_{\cal A} - \delta$. However, this rate might not be achievable. In order to show achievability then, we induce a memoryless channel by choosing for the control input a periodic sequence that ends with a reset symbol. More concretely, for *δ* > 0, the sequence consists of: a word with a value larger than $\mathrm{val}_{\cal A} - \delta$, a number of freeze symbols that guarantee an information rate larger than $\mathrm{val}_{\cal A} - 2\delta$ and a reset symbol. In the other direction, one would not expect a capacity larger than $\mathrm{val}_{\cal A}$. The reason is that the channel outputs a symbol uniformly at random when it is in a non-final state and this happens with probability at least $1 - \mathrm{val}_{\cal A}$.

Finally, note that at this point we do not know yet that the channel is information stable. Indeed, the proof of this fact (Corollary 1 in Supplementary Note 5) will use crucially Theorem 1.

### Formal statements of the main results

So far we have introduced the notion of uncomputable functions as those that cannot be computed with an algorithm (similarly the notion of undecidable problems). In order to make this definition, and hence the Main Results, mathematically rigorous, we have to recall the definition of a Turing Machine (TM) as the formal definition of what an algorithm is. For more details one can consult for instance^26,27^.

A TM represents a machine with a finite set of states that can read from and write to an infinitely long memory in the form of a tape. The tape is divided into cells that can hold a single symbol from a finite alphabet. Initially, the tape contains some arbitrary but finite string that we call the input followed by an infinite sequence of blank symbols. The operation of the machine is controlled by a head that sits on top of a cell of the tape. The head operates as follows: it reads the symbol below it; then, depending on the symbol and the current state it writes a symbol, moves left or right and transitions to a new state. The set of states includes the halting state. The TM halts after it transitions to the halting state. The output of the TM consists of the, possibly empty, string of symbols starting from the leftmost non-blank symbol to the rightmost non-blank symbol.

Formally, a TM is defined by a triple *M* = (*Q*,Σ,*δ*) where *Q* represents the finite set of states including an initial and a halting state, Σ is the finite set of symbols that a cell may contain and it includes the blank symbol and $\delta :(Q \times \Sigma ) \mapsto (Q \times \Sigma \times \{ L,R\} )$ is the transition function.

A configuration is a complete description of the status of a TM. It consists of the current state, the contents of the tape and the position of the head. In the initial configuration, the tape contains the input string and the head of the TM is in the initial state and situated on top of the leftmost cell of the input. Once the initial configuration is fixed a TM evolves deterministically and may or may not eventually halt.

Let us fix *n* = 10, *m* = 62. In order to specify an FSMC with *n* input symbols and *m* states, it is enough to give *N* = *nm*(2 + *m*) = 39,680 rational numbers corresponding to the conditional probability assignments. It is very easy to construct an injective map $\sigma ( \cdot )$ from vectors of *N* positive rational numbers to the natural numbers (see Supplementary Note 3), which then can be transformed into a valid input of a TM. For instance, it would be transformed into a string of zeroes and ones if Σ = {0, 1, #}. Main Results 1 and 2 can be then respectively restated as:

*Main Result 1:* There does not exist any TM that halts on all inputs of the form* σ*(**N**) for ${\mathbf{N}} \in {\cal S}_1$ and outputs a rational number* c *such that the capacity of **N** verifies

2 $$\left| {C({\mathbf{N}}) - c} \right| \le \frac{1}{5}.$$

*Main Result 2:* There does not exist any TM that halts on all inputs of the form *σ*(**N**) for ${\mathbf{N}} \in {\cal S}_\lambda$ and outputs 1 if the capacity of **N** ≥ *λ *and 0 if the capacity of **N** ≤ *λ*/2.

## Discussion

We have proven that no algorithm can exist that approximates the capacity for all information stable FSMCs to any desired precision.

Our construction builds directly on top of several strong undecidability results of PFAs. Recent developments underlying these results suggest that it should be possible to reduce the dimensions of our construction^28^. It is an interesting problem to find the minimal dimensions for which uncomputability holds.

It is important to notice also that the channels appearing in our construction have long-term memory. Combined with the known results for memoryless channels, this suggests the existence of a tradeoff between the time-scale of the memory of a channel and the efficiency to compute its capacity. Giving precise quantitative bounds in this direction is an interesting open question.

It is also worth exploring other problems that could be attacked with similar techniques. The proof technique can be extended to the capacities of quantum channels with memory implying an even stronger inapproximability result in that case. We will make the explicit analysis in a forthcoming paper. Similar long-term memory effects appear in other interesting situations, associated with other entropic quantities. One paradigmatic example is cryptography, where in order to analyse the security of the sequential use of a device, one needs to assume the worst case-scenario in which the adversary keeps memory of its past uses. Both in the classical and in the quantum case, the techniques of this paper could provide insights on the difficulty to provide optimal results in cryptographic settings.

Furthermore, our result connects with recent work regarding the different capacities of memoryless quantum channels^29,30^, showing some evidence that these capacities might be uncomputable. Also, memoryless zero error capacities, both classical and quantum, are known to have highly non-trivial behaviour^31–35^. Unfortunately, the techniques used here exploit directly the memory of the channel and hence cannot be directly applied to the memoryless capacities. The question is however of unquestionable interest.

## Methods

### Notation

We denote random variables by capital letters *X*, *Y*,..., sets and PFA—see below for the definition—by calligraphic capital letters ${\cal X},{\cal Y},...$, channels by capital bold face letters **X**, **Y**,..., and instances of random variables by lower case letters *x*, *y*,.... We denote vectors with the same convention, whenever confusion might arise a superscript indicates the number of components of the vector and a subscript the concrete component: *X*^*n*^ = (*X*_1_, *X*_2_,..., *X*_*n*_) or *x*^*n*^ = (*x*_1_, *x*_2_,..., *x*_*n*_). We indicate a consecutive subset of *n* components of the vector with subscript notation [*a*, *a* + *n*−1]: *x*_[*a*, *a*+*n*−1]_ = (*x*_*a*_, *x*_*a*+1_,..., *x*_*a*+*n*−2_, *x*_*a*+*n*−1_).

A vector is called a probability vector if all its entries are non-negative and add up to one. A matrix is called a stochastic matrix if all its columns are probability vectors. A stochastic matrix takes probability vectors to probability vectors (see Supplementary Note 1 for examples).

### Probabilistic finite automata

A PFA consists of a finite set of inputs and a finite set of states. One of these states is the initial state and a subset of the states are accepting states.

The action of the PFA is defined by the transition probabilities from one state to another as a function of the input symbols. A word is a sequence of symbols. After a word is fed to a PFA in the initial state, the PFA will transition from state to state and will end up in an accepting state with some probability. We call this probability the accepting probability of a word. Intuitively, we can understand a PFA as a machine with noisy knobs, the input symbols, and the input word is a sequence of knobs that tries to steer the machine into some desired state. See Fig. 1 for an example and Supplementary Note 1 for a formal definition.

Given some PFA, we denote by $\mathrm{val}_{\cal A}$ the supremum of the acceptance probabilities over all input words:

3 $$\mathrm{val}_{\cal A} = \mathop {{\sup }}\limits_{\mathbf{w}} \,\mathrm{val}({\cal A},{\mathbf{w}}),$$

where $\mathrm{val}({\cal A},{\mathbf{w}})$ denotes the value of ***w*** when input into the PFA ${\cal A}$ and the optimization runs over all words of finite length.

We consider two types of PFAs that we name as freezable and resettable.

We call a PFA a freezable PFA if one of the transition matrices is equal to the identity matrix ${\cal X}_{\mathrm{id}}$. The reason is that for such a PFA reading the symbol corresponding to the identity leaves the state probabilities unchanged. Let *u* be any probability vector, then

4 $$u = {\cal X}_{\mathrm{id}}u.$$

We call a PFA a resettable PFA if one of the transition matrices, ${\cal X}_{\mathrm{rt}}$, takes the state back to the initial state. Let *u* be any probability vector, then

5 $$v = {\cal X}_{\mathrm{rt}}u.$$

We let *γ* be a map from PFAs to PFAs such that for all PFA ${\cal A}$, $\gamma ({\cal A})$ is freezable and resettable. More concretely:

### Definition 1

Given a PFA ${\cal A} = \{ {\cal Q},{\cal W},{\cal X},v,{\cal F}\}$*,*we define $\gamma ({\cal A}) = \{ {\cal Q},{\cal W} \cup \{ \mathrm{id,rt}\} ,{\cal X} \cup \{ {\cal X}_{\mathrm{id}},{\cal X}_{\mathrm{rt}}\} ,v,{\cal F}\}$ as an automaton that extends ${\cal A}$ with the two additional input symbols id and rtand the corresponding matrices ${\cal X}_{\mathrm{id}}$ and ${\cal X}_{\mathrm{rt}}$ as given by (4) and (5).

The key lemma we will need about PFAs, essentially due to Gimbert and Oualhadj^25^, is the fact that their value cannot be approximated within a constant error. Let us give the precise statement. The complete proof can be found in Supplementary Note 6. Fix a rational number *λ* ∈ (0, 1].

### Lemma 1

*One can give explicitly a subfamily* ${\cal T}_\lambda$ *of rational freezable and resettable PFA with alphabet size* 5 *and* 62 *states with the following properties*:

(i) $\mathrm{val}_{\cal A}$ *is either* ≥λ *or* ≤λ/2 *for all* ${\cal A} \in {\cal T}_\lambda$.
(ii) *It is undecidable to know which is the case*.

The definition of ${\cal T}_\lambda$ will be given in Supplementary Note 6, Eq. (85).

### The family ${\cal S}_\lambda$ and the proof of Main Result 2

Given a freezable and resettable PFA ${\cal A}$ we define the channel ${\mathbf{V}}_{\cal A}$ as follows. The input alphabet of the channel takes values in $\{ 0,1\} \times {\cal W}$, which we identify with two different input registers: a data input and a control input. The data input is transmitted to the output: noiselessly if ${\cal A}$ is in an accepting state or, if ${\cal A}$ in any other state, the channel outputs uniformly at random an element of the output alphabet. More concretely, the output of the channel is defined by the following conditional probability:

6 $$p(y_n|x_n,s_{n - 1}) = \left\{ {\begin{array}{*{20}{l}} {\frac{1}{2}} \hfill & {{\mathrm {if}}\,s_{n - 1} \notin {\cal F}} \hfill \\ 1 \hfill & {{\mathrm {if}}\,s_{n - 1} \in {\cal F}\,{\mathrm {and}}\,y_n = x_n} \hfill \\ 0 \hfill & {{\mathrm{else}}} \hfill \end{array}} \right.$$

The control input is fed to ${\cal A}$, which begins in the initial state, and the state transition probabilities are dictated by the PFA:

7 $$p(s_n|c_n,s_{n - 1}) = \left\langle {\pi _{s_n},{\cal X}_{c_n}\pi _{s_{n - 1}}} \right\rangle .$$

We connect the properties of PFA with the capacity of FSMCs in the next Theorem:

### Theorem 1

*The capacity of* ${\mathbf{V}}_{\cal A}$ *is given by*

8 $$C({\mathbf{V}}_{\cal A}) = {\mathrm{val}}_{\cal A}.$$

We defer the proof to Supplementary Note 4.

The family ${\cal S}_\lambda$ in Main Result 2 is defined simply as

$${\cal S}_\lambda = \{ {\mathbf{V}}_{\cal A}:{\cal A} \in {\cal T}_\lambda \} .$$

with ${\cal T}_\lambda$ the family introduced in Lemma 1 and defined in Supplementary Note 6, Eq. (85).

Main Result 2 is then a trivial consequence of Lemma 1 and Theorem 1.

Furthermore one can leverage Theorem 1 to show that all the channels in ${\cal S}_\lambda$ are information stable:

### Corollary 1

*Given* ${\mathbf{V}}_{\cal A} \in {\cal S}_\lambda$, ${\mathbf{V}}_{\cal A}$ *is information stable*.

The proof will be given in Supplementary Note 5.

### Data availability

Data sharing not applicable to this article as no datasets were generated or analysed during the current study.

## Electronic supplementary material

Supplementary Information(PDF 385 kb)
