# Supplementary material for: Memory effects can make the transmission capability of a communication channel uncomputable
Source: Nat Commun. 2018 Mar 20;9:1149. doi: 10.1038/s41467-018-03428-0 (PMC5861076; doi:10.1038/s41467-018-03428-0)
Supplement: Supplementary file 1 — Supplementary Information(PDF 385 kb) [file 41467_2018_3428_MOESM1_ESM.pdf]

Supplementary Note 1. PFA

A PFA $\mathcal{A}$ is given by a tuple $\mathcal{A} = (\mathcal{Q}, \mathcal{W}, \mathcal{X}, v, \mathcal{F})$. $\mathcal{Q}$ denotes a finite set of states, $\mathcal{W}$ denotes a finite input alphabet, $\mathcal{X}$ denotes a finite set of stochastic matrices with cardinality equal to the cardinality of the input alphabet, v denotes an initial probability distribution over $\mathcal{Q}$ and $\mathcal{F} \subseteq \mathcal{Q}$ denotes a set of accepting states. We say that the PFA is *rational* if the coefficients of $\mathcal{X}$ and v are rational numbers. We will only consider rational PFA in the sequel.

The action of a PFA is defined by the transition probabilities from one state to another as a function of the input symbols. If the automaton is in the state q_a and reads the letter w it transitions to the state q_b with probability:

$$p\left[q_a \xrightarrow{w} q_b\right] = (X_w)_{q_b,q_a} \tag{1}$$

$$= \left\langle \pi_{\{q_b\}}, X_w \pi_{\{q_a\}} \right\rangle \tag{2}$$

where we denote by $\langle a, b \rangle$ the scalar product between vectors a and b and by $\pi_{\mathcal{X}}$ a vector with ones in the positions indicated by $\mathcal{X}$ and zeroes in the remaining positions.

We exploit the same notation for the probability that the automaton transitions from the state q_a to the state q_b after reading the word $\mathbf{w} = (w_1, \ldots, w_{|\mathbf{w}|}) \in \mathcal{W}^{|\mathbf{w}|}$:

$$p\left[q_a \xrightarrow{\mathbf{w}} q_b\right] = \left\langle \pi_{\{q_b\}}, X_{w_{|\mathbf{w}|}} \cdot \ldots \cdot X_{w_1} \pi_{\{q_a\}} \right\rangle \ . \tag{3}$$

More generally, if we have a probability distribution over the states given by the column vector x and the PFA reads the letter w then the new distribution over the states is given by $X_w x$. A particularly relevant probability is the probability that the automaton ends in an accepting state after reading some word $\mathbf{w}$. We call this probability the probability of accepting $\mathbf{w}$ or the value of $\mathbf{w}$. It can be computed

$$\mathrm{val}(\mathcal{A}, \mathbf{w}) = \left\langle \pi_{\mathcal{F}}, X_{w_{|\mathbf{w}|}} \cdot \ldots \cdot X_{w_1} v \right\rangle \ . \tag{4}$$

We call the value of $\mathcal{A}$, which we denote by $\mathrm{val}_{\mathcal{A}}$, the supremum of the acceptance probabilities over all input words:

$$\mathrm{val}_{\mathcal{A}} = \sup_{\mathbf{w} \in \mathcal{W}^*} \mathrm{val}(\mathcal{A}, \mathbf{w}) \tag{5}$$

where we denote by $\mathcal{W}^*$ the set of finite length words in $\mathcal{W}$.

Whenever possible, we will represent graphically the different automata constructions. We will follow the following conventions. A state is denoted by a circle. An accepting state is denoted by a circle with a double line around it. In all automata in this paper, the initial distribution will have one coefficient with weight one. We indicate the corresponding state with an arrow that does not come from any state.

We indicate with $\xrightarrow{w,p}$ that if the automaton reads the letter w it transitions from the origin of the arrow to the state pointed by the arrow with probability p. In order to avoid clutter, we simplify the notation in several cases. If we do not show transitions corresponding to all input symbols, the missing transitions correspond to self-loops with probability one. We drop the probability and just write $\overset{w}{\to}$ if a transition occurs with probability one. We drop the input symbol and just write $\overset{p}{\to}$ if all input symbols transition with the same probability.

Example 1. *Consider the PFA given in Supplementary Figure 1. The automaton in the figure has three states $\mathcal{Q} = \{q_1, q_2, q_3\}$, two input symbols $\mathcal{W} = \{a, b\}$, the initial state is q_1 and there is a single accepting state q_3. By looking at the figure we can construct the stochastic matrices:*

$$X_a = \begin{pmatrix} 0.5 & 1 & 0 \\ 0.5 & 0 & 0.5 \\ 0 & 0 & 0.5 \end{pmatrix} , \quad X_b = \begin{pmatrix} 0 & 0 & 0 \\ 0 & 1 & 0.5 \\ 1 & 0 & 0.5 \end{pmatrix} \tag{6}$$

Now, assume that we see the word $\mathbf{w} = baa$, we can easily compute its value:

$$\begin{pmatrix} 0 & 0 & 1 \end{pmatrix} X_b \cdot X_a \cdot X_a \begin{pmatrix} 1 \\ 0 \\ 0 \end{pmatrix} = 0.25 \tag{7}$$

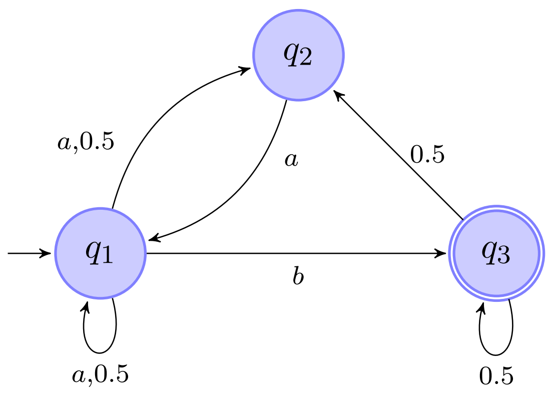

Supplementary Figure 1. Automaton with three states $\mathcal{Q} = \{q_1, q_2, q_3\}$, two input symbols $\mathcal{W} = \{a, b\}$, the initial state is q_1 and there is a single accepting state q_3.

Supplementary Note 2. FINITE STATE MACHINE CHANNELS

A channel can depend on past inputs and outcomes in very complicated ways. We focus our interest on finite FSMC which is the set of discrete channels that have its behavior dictated by a finite state machine [1]. Let $\mathcal{X}$, $\mathcal{Y}$ and $\mathcal{S}$ be finite sets that represent the input alphabet, output alphabet and set of states. A FSMC is characterized by the time-invariant conditional probabilities $p(y, s|x, s')$ for all states $s, s' \in \mathcal{S}$, input symbols $x \in \mathcal{X}$ and output symbols $y \in \mathcal{Y}$. These conditional probabilities denote the probability that the channel outputs the symbol y and transitions to the state s given that the channel is in state s' and receives the input symbol x. In this paper, we will restrict our attention to those FSMC for which $p(y, s|x, s')$ have a product form $p(y|x, s')p(s|x, s')$.

We assume that the initial state s_0 is known by both the transmitter and the receiver. We denote by $W^n_{s_0}$ the sequence of probability distributions induced by s_0 that give the probability of a sequence of outputs given a sequence of inputs into the channel:

$$W^n_{s_0}(y^n|x^n) = \sum_{s_n} W^n_{s_0}(y^n s_n|x^n) \tag{8}$$

where

$$\begin{aligned} W^n_{s_0}(y^n s_n|x^n) &= \\ &= \sum_{s_{n-1}} p(y_n, s_n|x_n, s_{n-1}) W^{n-1}_{s_0}(y_{n-1}s_{n-1}|x_{n-1}) \end{aligned} \tag{9}$$

Analogously we can define a sequence of probability distributions to characterize the state of the channel:

$$W^n_{s_0}(s_n|x^n) = \sum_{y^n} W^n_{s_0}(y^n s_n|x^n) \tag{10}$$

Note that we have used, abusing the notation, $W^n_{s_0}$ both to define the conditional probability of the output and the state. Consider two random variables X, Y with joint distribution $p(x, y)$, the information spectrum is the distribution of the random variable $i_{X,Y}$ given by:

$$i_{X,Y}(x, y) = \log \frac{p(y|x)}{p(y)} \tag{11}$$

The mutual information is the expected value of the information spectrum:

$$I(X;Y) = \langle i_{X,Y}(X, Y)\rangle_{XY} \tag{12}$$

$$= \sum_{x,y} p(x, y) \log \frac{p(y|x)}{p(y)} \tag{13}$$

A channel is said to be information stable [2] if for all $\gamma > 0$ there exists a sequence of random variables $\{X_i\}_{i=1}^{\infty}$ such that:

$$\lim_{n\to\infty} \Pr\left[\left|\frac{i_{X^n;W^n}(X^n;Y^n)}{nC^n} - 1\right| > \gamma\right] = 0 \tag{14}$$

where:

$$C_n = \sup_{X^n} \frac{1}{n} I(X^n;Y^n) \tag{15}$$

In full generality one may need to resort to the capacity formula of Verdu and Han [3] in order to compute the capacity of a FSMC. However, if the channel is information stable then the capacity is given by the mutual information rate [2]:

$$C(\mathbf{W}) = \lim_{n\to\infty} \sup_{X^n} \frac{1}{n} I(X^n;Y^n) \tag{16}$$

Supplementary Note 3. AN ENCODING FOR FSMC INTO TURING MACHINES

Let us construct an explicit example of the σ function introduced in the main text.

Since the set of FSMC can be seen as a subset of positive elements in $\mathbb{Q}^N$, it is enough to give an explicit injective map σ from the set of positive elements in $\mathbb{Q}^N$ into the natural numbers $\mathbb{N}$. For instance, consider the first $2N$ prime numbers $p_1, \ldots, p_{2N}$ and define

$$\sigma\left(\frac{r_1}{s_1}, \ldots, \frac{r_N}{s_N}\right) = \prod_{j=1}^{N} p_j^{r_j} \prod_{j=N+1}^{2N} p_j^{s_j} .$$

Any other explicit σ will do the job.

Supplementary Note 4. PROOF OF THEOREM 1

Proof. First we will prove that $\text{val}_{\mathcal{A}}$ is an achievable rate, that is: $C(\mathbf{V}_{\mathcal{A}}) \geq \text{val}_{\mathcal{A}}$. Then we will prove that $\text{val}_{\mathcal{A}}$ is an upper bound on the mutual information rate and in consequence $C(\mathbf{V}_{\mathcal{A}}) \leq \text{val}_{\mathcal{A}}$.

Let $\delta > 0$, then there exists some word $\mathbf{w}$ such that $\text{val}(\mathcal{A}, \mathbf{w}) \geq \text{val}_{\mathcal{A}} - \delta$, furthermore let $|\mathbf{w}| = m$. Consider the following protocol, the input into the control register is the deterministic sequence $(c_i)_{i=1}^{\infty}$ with

$$c_i = \begin{cases} w_i & \text{if } i-1 \mod m+n < m \\ \text{rt} & \text{if } i-1 \mod m+n = m+n-1 \\ \text{id} & \text{else} \end{cases} \tag{17}$$

This choice induces a memoryless channel when regarded in blocks of $m+n$ uses of the channel. That is, every block of $m+n$ inputs into the data input encounters exactly the same noisy channel once the control input is fixed by Supplementary Equation (17). In consequence, given this particular control input, any mutual information between the input and the output over $m+n$ uses is an achievable rate (once normalized over the number of uses). For the data input, we choose the uniform distribution.

The following chain of inequalities holds for the conditional entropy of the output given the input:

$$\begin{aligned} & H\left(Y_{[1,m+n]}|X_{[1,m+n]}C_{[1,m+n]}\right) \\ &= \sum_{i=1}^{m+n} H\left(Y_i|Y_{[1,i-1]}X_{[1,m+n]}C_{[1,m+n]}\right) && (18)\\ &\leq m + H\left(Y_{[m+1,m+n]}|Y_{[1,m]}X_{[1,m+n]}C_{[1,m+n]}\right) && (19)\\ &\leq m + H\left(Y_{[m+1,m+n]}|X_{[1,m+n]}C_{[1,m+n]}\right) && (20)\\ &\leq m + 1 + (1 - \text{val}_{\mathcal{A}} + \delta)n && (21) \end{aligned}$$

where Supplementary Equation (18) follows by the chain rule, the inequality Supplementary Equation (19) by bounding the entropy of the first m uses by m, the inequality Supplementary Equation (20) by removing the conditioning on $Y_{[1,m]}$ and

Supplementary Equation (21) holds from bounding the conditional entropy by Supplementary Equation (25) that we prove below.

After the first m uses, the automaton behaves like a noiseless channel with probability at least $\mathrm{val}_{\mathcal{A}} - \delta$ and like a completely random channel with the complementary probability. In consequence, we can bound the conditional entropy of the output of the uses $m+1$ to $m+n$ as follows:

$$H\big(Y_{[m+1,m+n]}|X_{[1,m+n]}C_{[1,m+n]}\big) \tag{22}$$
$$\leq H\big((\mathrm{val}_{\mathcal{A}} - \delta + (1-\mathrm{val}_{\mathcal{A}}+\delta)2^{-n})\phi + (1-\mathrm{val}_{\mathcal{A}}+\delta)(1-2^{-n})\rho\big) \tag{23}$$
$$= h(\mathrm{val}_{\mathcal{A}} - \delta + (1-\mathrm{val}_{\mathcal{A}}+\delta)2^{-n}) + (1-\mathrm{val}_{\mathcal{A}}+\delta)(1-2^{-n})\log(2^n-1) \tag{24}$$
$$\leq 1 + (1-\mathrm{val}_{\mathcal{A}}+\delta)n \tag{25}$$

where $\phi = (1, 0, \ldots, 0)$ is a completely deterministic probability vector of length 2^n, $\rho = \left(0, \frac{1}{2^n-1}, \frac{1}{2^n-1}, \ldots, \frac{1}{2^n-1}\right)$ the maximally entropic vector of length $2^n - 1$ and $h(\epsilon) := -\epsilon\log\epsilon - (1-\epsilon)\log(1-\epsilon)$ is the binary entropy function.

Now we can use Supplementary Equation (21) to bound the mutual information of the first $m+n$ uses:

$$I(Y^{m+n}; X^{m+n}C^{m+n}) \tag{26}$$
$$= H(Y^{m+n}) - H(Y^{m+n}|X^{m+n}C^{m+n}) \tag{27}$$
$$= m + n - H(Y^{m+n}|X^{m+n}C^{m+n}) \tag{28}$$
$$\geq n(\mathrm{val}_{\mathcal{A}} - \delta) - 1 \tag{29}$$

Finally, by choosing n larger than $(1 + (\mathrm{val}_{\mathcal{A}} - 2\delta)m)/\delta$ we get

$$\frac{1}{m+n} I(Y^{m+n}; X^{m+n}C^{m+n}) \geq \mathrm{val}_{\mathcal{A}} - 2\delta \tag{30}$$

That is, for all $\delta > 0$ the rate $\mathrm{val}_{\mathcal{A}} - 2\delta$ is achievable.

Now we will prove that $C(\mathbf{V}_{\mathcal{A}})$ is upper bounded by $\mathrm{val}_{\mathcal{A}}$.

Let S_i denote the state of the PFA at use i, since the output only depends on the control input through the PFA state we have that $H(Y_i|X_iC_{[1,i-1]}) \geq H(Y_i|X_iS_{i-1})$. In consequence, we can bound from below the conditional entropy of the output given the input as follows:

$$H\big(Y^n|X^nC^n\big)$$
$$= \sum_{i=1}^{n} H\left(Y_i|Y_{[1,i-1]}X^nC^n\right) \tag{31}$$
$$= \sum_{i=1}^{n} H\left(Y_i|Y_{[1,i-1]}X_iC_{[1,i-2]}\right) \tag{32}$$
$$\geq \sum_{i=1}^{n} H(Y_i|S_{i-1}X_i) \tag{33}$$
$$= \sum_{i=1}^{n} p(S_{i-1} \in \mathcal{F})H(Y_i|S_{i-1} \in \mathcal{F}, X_i = 0) + p(S_{i-1} \notin \mathcal{F})H(Y_i|S_{i-1} \notin \mathcal{F}, X_i = 0) \tag{34}$$
$$\geq n(1 - \mathrm{val}_{\mathcal{A}}) \tag{35}$$

Finally, we can plug the bound on the conditional entropy to obtain the desired result:

$$\frac{1}{n} I(Y^n; X^nC^n) = \frac{1}{n}(H(Y^n) - H(Y^n|X^nC^n)) \tag{36}$$
$$\leq 1 - \frac{1}{n} H(Y^n|X^nC^n) \tag{37}$$
$$\leq \mathrm{val}_{\mathcal{A}} \tag{38}$$

□

Supplementary Note 5. PROOF OF COROLLARY 1

Proof. From the proof of Theorem 1 we know that $\forall \delta > 0, \forall t \in \mathbb{N}$ there exists n_t (wlog $n_{t+1} \geq n_t$) and $X^t = \{X_1, \ldots, X_{n_t}\}$ such that:

$$\frac{I(X^t;Y^t)}{n_t} \geq \mathrm{val}_{\mathcal{A}} - \frac{\delta}{2^t} \tag{39}$$

We define the following source to input into the channel:

$$\mathbf{V} = \left\{ \underbrace{X^1, \ldots, X^1}_{m_1 \text{ times}}, \ldots, \underbrace{X^t, \ldots, X^t}_{m_t \text{ times}}, \ldots \right\} \tag{40}$$

Each use of the channel is uniquely identified by a triple (t, α, β) with $t \in \mathbb{N}$, $\alpha \in [0, m_{t+1} - 1]$ and $\beta \in [0, n_{t+1}]$ such that the triple corresponds with the use n-th with

$$n = \sum_{i=1}^{t} m_i n_i + \alpha n_{t+1} + \beta \tag{41}$$

and the sequence of random variables that is input over the first n uses is

$$V^n = \tag{42}$$
$$= \left\{ \underbrace{X^1, \ldots, X^1}_{m_1 \text{ times}}, \ldots, \underbrace{X^t, \ldots, X^t}_{m_t \text{ times}}, \underbrace{X^{t+1}, \ldots, X^{t+1}}_{\alpha \text{ times}}, X^{t+1}_{[1,\beta]} \right\}$$

The sequence $\{m_i\}_{i=1}^{\infty}$ is chosen such that:

$$\frac{I\left(V^n;W^n\right)}{n} \geq \mathrm{val}_{\mathcal{A}} - \frac{\delta}{2^{t-1}} \tag{43}$$

where W^n is the random variable induced by V^n at the output of the channel and n is related to t by Supplementary Equation (41).

Let $C_n = \sup_{X^n} I(X^n;Y^n)/n$, Supplementary Equation (43) implies that

$$1 \geq \mathbb{E}\left[\frac{i_{V^n,W^n}}{nC_n}\right] \geq \mathbb{E}\left[\frac{i_{V^n,W^n}}{n\mathrm{val}_{\mathcal{A}}}\right] \geq 1 - \frac{\delta}{\mathrm{val}_{\mathcal{A}} 2^{t-1}} \tag{44}$$

Note that the input V^n is composed of independent random variables, hence:

$$i_{V^n,W^n} = \sum_{i=1}^{t}\sum_{j=1}^{m_i} i_{X^i,Y^i} + \sum_{i=1}^{\alpha} i_{X^{t+1},Y^{t+1}} + i_{X^{t+1}_{[1,\beta]},Y^{t+1}_{[1,\beta]}} \tag{45}$$

and also note that for all t and $\beta \in [0, n_t]$:

$$0 \leq i_{X^t_{[1,\beta]},Y^t_{[1,\beta]}} \leq \beta \tag{46}$$

In order to achieve Supplementary Equation (43), we see how m_t can be chosen:

$$\begin{aligned} I(V^n;W^n) &= \sum_{i=1}^{t-1} m_i I(X^i;Y^i) + m_t I(X^t;Y^t) \\ &\quad + \alpha I(X^{t+1};Y^{t+1}) + I(X^{t+1}_{[1,\beta]};Y^{t+1}_{[1,\beta]}) \qquad (47) \\ &\geq \sum_{i=1}^{t} m_i n_i \left(\mathrm{val}_{\mathcal{A}} - \frac{\delta}{2^i}\right) + \alpha n_{t+1}\left(\mathrm{val}_{\mathcal{A}} - \frac{\delta}{2^{t+1}}\right) \qquad (48) \end{aligned}$$

In order to verify Supplementary Equation (43) it suffices to choose m_t larger than

$$\left\lceil \frac{2^t}{n_t\delta}\left(\sum_{i=1}^{t-1} m_i n_i \delta\left(\frac{1}{2^i}-\frac{1}{2^{t-1}}\right)+n_{t+1}\left(\mathrm{val}_{\mathcal{A}}-\frac{\delta}{2^{t-1}}\right)\right)\right\rceil \tag{49}$$

such that the following holds

$$\sum_{i=1}^{t} m_i n_i\left(\mathrm{val}_{\mathcal{A}}-\frac{\delta}{2^i}\right) \geq \left(\sum_{i=1}^{t} m_i n_i + n_{t+1}\right)\left(\mathrm{val}_{\mathcal{A}}-\frac{\delta}{2^{t-1}}\right) \tag{50}$$

However, for technical reasons in the concentration bounds that follow we choose:

$$m_t = \max\left\{\text{Suplementary Equation (49)}, (n_{t+1})^2\right\} \tag{51}$$

In the following we prove that $\forall \eta > 0$:

$$\lim_{n\to\infty} \Pr\left[\left|\frac{i_{V^n,W^n}}{nC_n}-1\right| \geq \eta\delta\right] = 0 \tag{52}$$

Let us expand the probability expression in Supplementary Equation (52):

$$\begin{aligned}
&\Pr\left[\left|\frac{i_{V^n,W^n}}{nC_n}-1\right| \geq \eta\delta\right] = \\
&\quad= \Pr\left[\frac{i_{V^n,W^n}}{nC_n}-1 \geq \eta\delta\right] + \Pr\left[\frac{i_{V^n,W^n}}{nC_n}-1 \leq -\eta\delta\right] && (53)\\
&\quad\leq \Pr\left[\frac{i_{V^n,W^n}}{nC_n}-\mathbb{E}\left[\frac{i_{V^n,W^n}}{nC_n}\right] \geq \eta\delta\right] \\
&\qquad+ \Pr\left[\frac{i_{V^n,W^n}}{nC_n}-\mathbb{E}\left[\frac{i_{V^n,W^n}}{nC_n}\right] \leq -\eta\delta + \frac{\delta}{\mathrm{val}_{\mathcal{A}}2^{t-1}}\right] && (54)\\
&\quad\leq \Pr\left[\left|i_{V^n,W^n}-\mathbb{E}\left[i_{V^n,W^n}\right]\right| \geq nC_n\delta\left(\eta-\frac{1}{\mathrm{val}_{\mathcal{A}}2^{t-1}}\right)\right] && (55)
\end{aligned}$$

Now we will exploit that i_{V^n,W^n} can be expressed as a sum of $l = \sum_{i=1}^{t} m_i + \alpha + 1$ independent random variables (see Supplementary Equation (41)). For these sums we can bound the two-tailed probability via Hoeffding's inequality [4]. More concretely, let $\{X_i\}_{i=1}^{l}$ be a sequence of l independent random variables, let $t \geq 0$ and let $a_i \leq X_i \leq b_i$ then:

$$\Pr\left[\left|\sum_{i=1}^{l} X_i - \mathbb{E}\left[\sum_{i=1}^{l} X_i\right]\right| \geq t\right] \leq 2\exp\left(\frac{-2t^2}{\sum_{i=1}^{l}|b_i-a_i|^2}\right) \tag{56}$$

We can make clearly the identifications with Supplementary Equation (55) and Supplementary Equation (45). However, before applying Hoeffding's inequality let us bound the denominator in the exponential term:

$$\begin{aligned}
\sum_{i=1}^{l}|b_i-a_i|^2 &= \sum_{i=1}^{t} m_i(n_i)^2 + \alpha(n_{t+1})^2 + \beta^2 && (57)\\
&\leq \sum_{i=1}^{t} m_i n_i n_{t+1} + \alpha n_{t+1} n_{t+1} + \beta n_{t+1} && (58)\\
&\leq n^{3/2} && (59)
\end{aligned}$$

The last inequality follows because from Supplementary Equation (51) we have that $(n_{t+1})^2 \leq m_t \leq n$. Now if we apply Hoeffding's inequality to Supplementary Equation (55) we can bound it from above by:

$$2\exp\left(-\frac{2\left(nC_n\delta\left(\eta-\frac{1}{\mathrm{val}_{\mathcal{A}}2^{t-1}}\right)\right)^2}{n^{3/2}}\right) \tag{60}$$

and in consequence the limit when n goes to infinity is zero for all $\eta > 0$. □

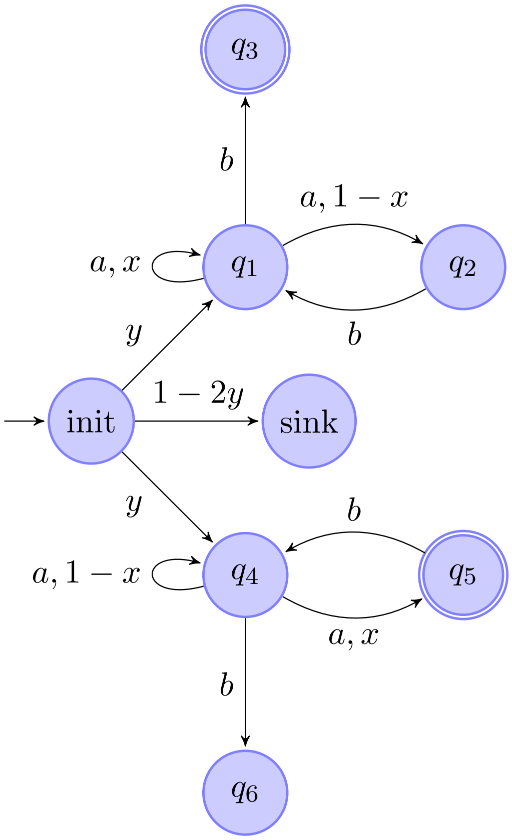

Supplementary Figure 2. The automaton $\mathcal{D}_{x,y}$ has value $2y$ if $x > 1/2$ and value $\leq y$ if $x \leq 1/2$.

Supplementary Note 6. PROOF OF LEMMA 1

Lemma 1 is essentially proven by Gimbert and Oualhadj in [5] with a very elegant construction (a succinct sketch can be found in [6]). We include a full proof here for completeness, to cover the case of an arbitrary λ (in [5] they only consider the case $\lambda = 1$) and to include in the construction an undecidability result of Hirvensalo [7]. This allows us to give the concrete estimates of alphabet size 10 and 62 states that appear in Lemma 1.

A. The construction of Gimbert and Oualhadj

Lemma 1 (Proposition 5 [6]). *Let $\mathcal{D}_{x,y}$ be the automaton in Supplementary Figure 2 and $x \in [0,1]$, $y \in [0,1/2]$. $\mathcal{D}_{x,y}$ has value $2y$ if $x > 1/2$ and value $\leq y$ if $x \leq 1/2$.*

Proof. First, we need to make some observations regarding $\mathcal{D}_{x,y}$. If the input letter b is fed two or more consecutive times the automaton is forced it into the states sink, q_3 and q_6 from which the automaton cannot exit. For any such a word, the acceptance value is y. Hence, we concentrate our attention to words of the form $a^{n_1}ba^{n_2}b\dots ba^{n_t}b$. For any word $\mathbf{w}$ of this form the acceptance value is:

$$\mathrm{val}(\mathcal{A}, w) = y\,p\left[q_1 \xrightarrow{\mathbf{w}} q_3\right] + y\,p\left[q_4 \xrightarrow{\mathbf{w}} q_5\right] \tag{61}$$

$$\leq y\,p\left[q_1 \xrightarrow{\mathbf{w}} q_3\right] + y\left(1 - p\left[q_4 \xrightarrow{\mathbf{w}} q_6\right]\right) \tag{62}$$

Furthermore, the upper bound is reachable. To verify this, consider the word $\mathbf{w}a^n$, $p\left[q_1 \xrightarrow{\mathbf{w}a^n} q_3\right]$ does not change and we can make $p\left[q_4 \xrightarrow{\mathbf{w}a^n} q_5\right]$ approach $1 - p\left[q_4 \xrightarrow{\mathbf{w}a^n} q_6\right]$ by choosing n large enough. Both $p\left[q_1 \xrightarrow{\mathbf{w}} q_3\right]$ and $p\left[q_4 \xrightarrow{\mathbf{w}} q_6\right]$ admit

a very compact form:

$$p\left[q_1 \xrightarrow{\mathbf{w}} q_3\right] = 1 - \prod_{i=1}^{t}(1 - x^{n_i}) \tag{63}$$

$$p\left[q_4 \xrightarrow{\mathbf{w}} q_6\right] = 1 - \prod_{i=1}^{t}(1 - (1-x)^{n_i}) \tag{64}$$

Let us consider first $x \leq 1/2$. This implies that $x \leq 1 - x$ and in consequence

$$1 - \prod_{i=1}^{t}(1 - x^{n_i}) \leq 1 - \prod_{i=1}^{t}(1 - (1-x)^{n_i}) \ . \tag{65}$$

Let $\epsilon > 0$, for any word $\mathbf{w}$ such that $p[q_1 \to q_3] = 1 - \epsilon$ we have $p[q_4 \to q_6] \geq 1 - \epsilon$ and $\mathrm{val}(\mathcal{D}_{x,y}, \mathbf{w}) \leq y$.

Let us assume now that $x > 1/2$. We are going to prove that for any $\epsilon \in (0, x)$ there exists a word $\mathbf{w}$ such that:

$$p\left[q_4 \xrightarrow{\mathbf{w}} q_6\right] \leq \epsilon \tag{66}$$

$$p\left[q_1 \xrightarrow{\mathbf{w}} q_3\right] \geq 1 - \epsilon \tag{67}$$

Consider the sequence of words $\{\mathbf{w_k}\}_{k=2}^{\infty}$ where $\mathbf{w_k} = a^{n_2} b a^{n_3} n \dots b a^{n_k}$ and the lengths $n_2 \dots n_k$ are given by

$$n_k = \left\lceil \log_x \frac{1}{k} + C_\epsilon \right\rceil \tag{68}$$

and

$$C_\epsilon = \frac{1}{b} \log_x \left(\frac{b-1}{b} \epsilon \right) \ . \tag{69}$$

Let $b > 1$ be a number such that $x^b = 1 - x$. The following sequence of inequalities holds:

$$\begin{aligned} p\left[q_4 \xrightarrow{\mathbf{w_k}} q_6\right] &= (1-x)^{n_1} + (1 - (1-x)^{n_2})(1-x)^{n_3} + \dots \\ &\quad + \prod_{i=2}^{k-1}(1 - (1-x)^{n_i})(1-x)^{n_k} && (70) \\ &\leq \sum_{i=2}^{k}(1-x)^{n_i} && (71) \\ &= \sum_{i=2}^{k} x^{b n_i} && (72) \\ &= \sum_{i=2}^{k} x^{b \lceil \log_x \frac{1}{i} + C_\epsilon \rceil} && (73) \\ &\leq x^{b C_\epsilon} \sum_{i=2}^{k} x^{b \log_x \frac{1}{i}} && (74) \\ &= x^{b C_\epsilon} \sum_{i=2}^{k} \frac{1}{i^b} && (75) \end{aligned}$$

Note that the sum in the right hand side of Supplementary Equation (75) when k goes to infinity is very similar to the Riemann zeta function evaluated at a real argument strictly larger than one. For these arguments it is well known [8] that it can be bounded by

$$\zeta(b) = \sum_{n=1}^{\infty} \frac{1}{n^b} \leq \frac{b}{b-1} \ . \tag{76}$$

If we apply this bound to Supplementary Equation (75) we obtain

$$\lim_{k\to\infty} p\left[q_4 \xrightarrow{\mathbf{w_k}} q_6\right] \leq \lim_{k\to\infty} x^{bC_\epsilon}\sum_{i=2}^{k}\frac{1}{i^b} \quad (77)$$

$$\leq x^{bC_\epsilon}\frac{b}{b-1} \quad (78)$$

$$= \epsilon\ . \quad (79)$$

Furthermore, Supplementary Equation (79) remains an upper bound for finite k since we are only dropping positive contributions. Hence, Supplementary Equation (66) is verified for all k. Let us now verify that there exists k such that the requirement Supplementary Equation (67) also holds. Consider the following sum

$$\sum_{i=2}^{k} x^{n_i} \geq \sum x^{\log_x \frac{1}{i}+C_\epsilon+1} \quad (80)$$

$$= x^{C_\epsilon+1}\sum_{i=2}^{k}\frac{1}{i} \quad (81)$$

and this sum diverges for any non-zero x and finite C_ϵ. This implies that $\lim_{k\to\infty}\prod_{i=2}^{k}(1-x^{n_i}) = 0$ and that there exists a finite k such that $\prod_{i=2}^{k}(1-x^{n_i}) \leq \epsilon$. Then, $p\left[q_1 \xrightarrow{\mathbf{w_k}} q_3\right] \geq 1-\epsilon$. □

Now, we are going to modify $\mathcal{D}_{x,y}$. The main idea is that x will be replaced by the probability that an automaton $\mathcal{A}$ accepts a word $\mathbf{w}_\mathcal{A}$. This is achieved very easily, see Supplementary Figure 3, once the state of $\mathcal{D}_{\mathcal{A},y}$ reaches $\mathcal{A}$ it continues inside the automaton until it sees c which is a symbol outside the input alphabet of $\mathcal{A}$. Then, it will transition to one of two different states depending on whether or not $\mathcal{A}$ is in an accepting state. We indicate the transitions from an accepting state by $\Longrightarrow$ and the transitions from a non-accepting state by $\dashrightarrow$. Let $\mathbf{w}_\mathcal{A}$ be an arbitrary input word into $\mathcal{A}$ then:

$$p\left[q_1 \xrightarrow{a\mathbf{w}_\mathcal{A}c} q_1\right] = \text{val}(\mathcal{A},\mathbf{w}_\mathcal{A}) \quad (82)$$

$$p\left[q_4 \xrightarrow{a\mathbf{w}_\mathcal{A}c} q_5\right] = \text{val}(\mathcal{A},\mathbf{w}_\mathcal{A}) \quad (83)$$

In the following we reduce the problem of finding the value of $\mathcal{D}_{\mathcal{A},y}$ to the emptiness of the set $L_{\mathcal{A}>\lambda}$. This is the set of words with acceptance probability strictly higher than λ. That is: $L_{\mathcal{A}>\lambda} = \{\mathbf{w}\in\mathcal{W}^* : \text{val}(\mathcal{A},\mathbf{w}) > \lambda\}$.

Lemma 2. *Given a PFA $\mathcal{A}$ and $y\in[0,1/2]$, the automaton $\mathcal{D}_{\mathcal{A},y}$ has value $\leq y$ if $L_{\mathcal{A}>1/2}$ is empty and value $\geq 2y$ if not.*

Proof. Assume first that $L_{\mathcal{A}>1/2}$ is not empty. Then there exists some $\mathbf{w}_\mathcal{A}$ such that $\text{val}(\mathcal{A},\mathbf{w}_\mathcal{A}) > 1/2$. Hence, we can construct the sequence $\mathbf{w_k} = (a\mathbf{w}_\mathcal{A}c)^{n_2}\dots(a\mathbf{w}_\mathcal{A}c)^{n_k}$ with the lengths $n_2\dots n_k$ given by Supplementary Equation (68). Following the proof of Lemma 1 we have that for $\epsilon > 0$ there exists k such that $\mathbf{w_k}$ verifies conditions Supplementary Equation (66) and Supplementary Equation (67).

Assume now that $L_{\mathcal{A}>1/2}$ is empty. We can restrict our attention to words of the form $(a\mathbf{w}^1_\mathcal{A}c)^{n_1}b\dots b(a\mathbf{w}^k_\mathcal{A}c)^{n_k}b$. Furthermore for any word $\mathbf{w}$ we have that $\text{val}(\mathcal{A},\mathbf{w}) \leq 1-\text{val}(\mathcal{A},\mathbf{w})$ and in consequence

$$1-\prod_{i=1}^{k}\left(1-\text{val}(\mathcal{A},\mathbf{w}^i_\mathcal{A})^{n_i}\right) \leq 1-\prod_{i=1}^{k}\left(1-\left(1-\text{val}(\mathcal{A},\mathbf{w}^i_\mathcal{A})^{n_i}\right)\right) \quad (84)$$

Let $\epsilon > 0$ Supplementary Equation (84) implies that for any word such that $p\left[q_1 \xrightarrow{\mathbf{w}} q_4\right] = 1-\epsilon$ we have that $p\left[q_4 \xrightarrow{\mathbf{w}} q_6\right] \geq 1-\epsilon$ and $\text{val}(\mathcal{D}_{\mathcal{A},y},\mathbf{w}) \leq y$. □

Let us close this section by defining the family $\mathcal{T}_\lambda$ as

$$\mathcal{T}_\lambda = \left\{\gamma(\mathcal{D}_{\mathcal{A},\lambda/2}) : \mathcal{A}\text{ has a binary alphabet and 27 states}\right\} \quad (85)$$

with γ as in Definition 1.

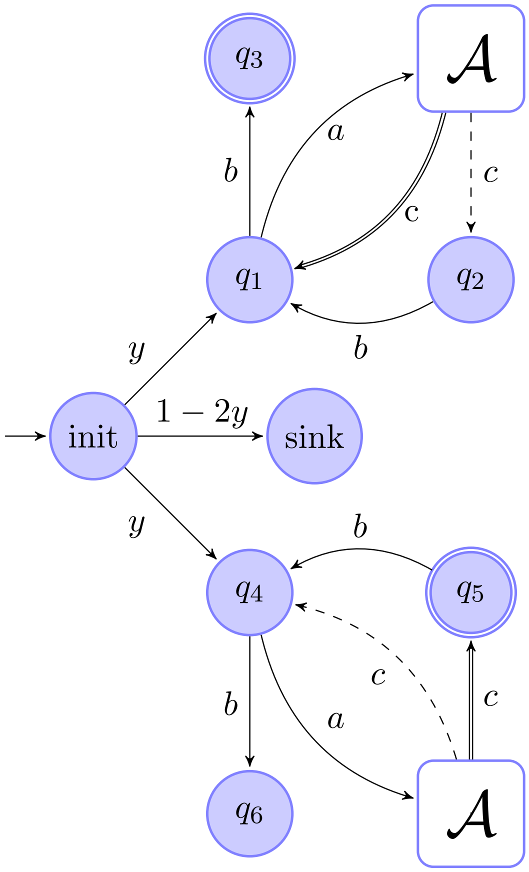

Supplementary Figure 3. The automaton $\mathcal{D}_{\mathcal{A},y}$ has value $\leq y$ if $L_{\mathcal{A}>1/2}$ is empty and value $\geq 2y$ if not.

B. The undecidability result of Hirvensalo

To use the above construction in order to prove Lemma 1, up to the issue of restricting to freezable and resettable channels (that we will take care of below), it only remains to show that deciding whether $L_{\mathcal{A}>1/2}$ is empty or not is indeed undecidable. This problem, known as the emptiness problem, was proved undecidable in [9–11]. Recently, new proofs with explicit bounds in the number of states and the cardinality of the alphabet have been derived in [6, 7, 12] together with an undecidability proof of several related sets. Here, we will rely on

Theorem 1 ([7]). *Let k be an integer equal or greater than 7 and (n, m) be a duple of integers that is equal or pointwise larger than $(2, 5k-10)$. The emptiness of $L_{\mathcal{A}>\delta}$, for $\delta = 1/(5k-10)$ and PFAs with alphabet size n and m states, is undecidable.*

Taking Theorem 1 as a starting point, we can amplify the result and obtain undecidability for any rational $\delta \in (0, 1)$ (in particular for $\delta = 1/2$).

Corollary 1. *Fix any rational number δ. The emptiness of $L_{\mathcal{A}>\delta}$ for PFAs with alphabet size 2 and 27 states is undecidable.*

Proof. Given an arbitrary PFA $\mathcal{A} = (\mathcal{Q}, \mathcal{W}, \mathcal{X}, v, \mathcal{F})$ and $p \in (0, 1)$ we are going to construct two PFAs $\mathcal{B}_p$ and $\mathcal{C}_p$ such that: $L_{\mathcal{A}>\delta}$ is empty $\Leftrightarrow L_{\mathcal{B}_p>p\delta}$ is empty $\Leftrightarrow L_{\mathcal{C}_p>p\delta+1-p}$ is empty.

Let us first construct $\mathcal{B}_p = (\mathcal{T}, \mathcal{W}, \mathcal{Y}, u, \mathcal{F})$. The set of states is $\mathcal{T} = \{\mathcal{Q} \cup \text{init} \cup \text{sink}\}$. The input alphabet is equal to the original one. For any input symbol $x \in \mathcal{W}$ we define the stochastic matrices of $\mathcal{B}_p$ as follows:

$$Y_x = \left(\begin{array}{ccc|cc} & & & & 0 \\ & X_x & & p\,X_x\,v & \vdots \\ & & & & 0 \\ \hline 0 & \dots & 0 & 0 & 0 \\ 0 & \dots & 0 & \text{1-p} & 1 \end{array}\right) \tag{86}$$

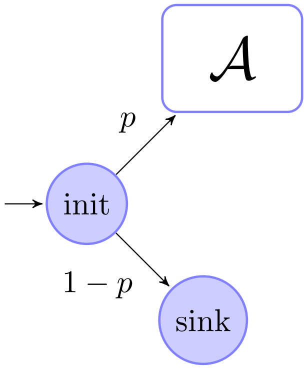

(a) $\mathcal{B}_p$

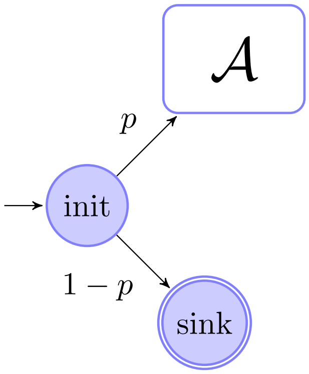

(b) $\mathcal{C}_p$

Supplementary Figure 4. The automata $\mathcal{B}_p$ (left) and $\mathcal{C}_p$ (right) can be used to amplify the undecidability of the emptiness problem to arbitrary $\delta \in (0,1)$.

Note that we have added two rows and columns to track the two new states. Let us parse the action of the automaton as defined by the stochastic matrices. If it is in any of the original states, its behavior remains unchanged. If the automaton is in the sink state no matter what input symbol it reads the PFA remains in the sink state. Finally, if the automaton is in the init state upon reading the input symbol x with probability $1-p$ it will transition to the sink state and with probability p it will transition to whatever the original automaton would have transitioned from the initial distribution. In other words, the new distribution on the states will be given by $(p\, X_x\, v, 0, 1-p)$. The initial distribution of $\mathcal{B}_p$ has weight one on the init state, that is: $u = (0, \ldots, 0, 1, 0)$.

The construction of $\mathcal{C}_p$ is identical except that we add the sink state to the set of accepting states. We have depicted both constructions in Supplementary Figure 4.

For any input word $\mathbf{w} \in \mathcal{W}^*$ we have that $\mathrm{val}(\mathcal{A}, \mathbf{w}) = p\,\mathrm{val}(\mathcal{B}_p, \mathbf{w}) = p\,\mathrm{val}(\mathcal{C}_p, \mathbf{w}) + 1 - p$. Hence, $L_{\mathcal{A}>\delta}$ is empty $\Leftrightarrow$ $L_{\mathcal{B}_p>p\delta}$ is empty $\Leftrightarrow$ $L_{\mathcal{C}_p>p\delta+1-p}$ is empty. $\square$

C. Resettable and freezable channels

By the definition of the family $\mathcal{T}_\lambda$ given in (85), Lemma 1 is just a consequence of Lemma 2, Corollary 1 and the following lemma, whose proof finishes the paper.

Lemma 3. *$val_{\tilde{\mathcal{A}}} = val_{\gamma(\tilde{\mathcal{A}})}$ for all PFA $\tilde{\mathcal{A}}$ of the form $D_{\mathcal{A},y}$.*

Proof. Given a PFA $\mathcal{A}$, we define the set $\mathrm{values}(\mathcal{A}) = \{\mathrm{val}(\mathcal{A}, \mathbf{w}) | \mathbf{w} \in \mathcal{W}^*\}$. This is the set of achievable values or, alternatively, it can be regarded as the range of the function $\mathrm{val}(\mathcal{A}, \mathbf{w})$ once the PFA $\mathcal{A}$ is fixed. It is then enough to show that $\mathrm{values}(\tilde{\mathcal{A}}) = \mathrm{values}(\gamma(\tilde{\mathcal{A}}))$ for any PFA $\tilde{\mathcal{A}}$ of the form $D_{\mathcal{A},y}$.

$\supseteq$

This direction is trivial since any input word $\mathbf{w}$ of $\tilde{\mathcal{A}}$ is also an input word of $\gamma(\tilde{\mathcal{A}})$ and $\mathrm{val}(\gamma(\tilde{\mathcal{A}}), \mathbf{w}) = \mathrm{val}(\tilde{\mathcal{A}}, \mathbf{w})$.

$\subseteq$

Let us divide the input words into two sets: W_1 the words that either end with the symbol rt or consist of a string of id and W_2 which is the complementary set, that is, words that have at least one symbol different than id and do not end with the rt symbol. The acceptance probability of any $\mathbf{w} \in W_1$ is simply the acceptance probability of a distribution with unit probability on the initial symbol. Since for $\mathcal{D}_{\tilde{\mathcal{A}},y}$ the acceptance and initial symbols are disjoint, the value of $\mathbf{w}$ is zero. That means that no word from W_1 can be in the set $\{\mathbf{w} : \mathrm{val}(\gamma(\tilde{\mathcal{A}}), \mathbf{w}) \geq \lambda\}$ for any value of $\lambda \in (0, 1]$.

First, consider any word $\mathbf{w} \in W_2$ that contains at least one identity symbol, it can be written as $\mathbf{w_1}\mathrm{id}\mathbf{w_2}$ where $\mathbf{w_1}$ and $\mathbf{w_2}$ are two sequences of input symbols and at least one of both is non empty. We have that $\mathrm{val}(\gamma(\tilde{\mathcal{A}}), \mathbf{w}) = \mathrm{val}(\gamma(\tilde{\mathcal{A}}), \mathbf{w_1}\mathbf{w_2})$ and by applying this argument to all the identity symbols in the word we find a new word $\mathbf{w}'$ with no identity symbols such that $\mathrm{val}(\gamma(\tilde{\mathcal{A}}), \mathbf{w}) = \mathrm{val}(\gamma(\tilde{\mathcal{A}}), \mathbf{w}')$. Hence we can restrict our attention to words with no identity symbol.

Second, we consider any word $\mathbf{w} \in W_2$ that contains at least one reset symbol, it can be written as $\mathbf{w_1}\mathrm{rt}\mathbf{w_2}$ where at least $\mathbf{w_2}$ is non empty. We have that $\mathrm{val}(\gamma(\tilde{\mathcal{A}}), \mathbf{w}) = \mathrm{val}(\gamma(\tilde{\mathcal{A}}), \mathbf{w_2})$, again we can apply this argument to all the reset symbols in the word and find a word $\mathbf{w}'$ with no reset or identity symbols such that $\mathrm{val}(\gamma(\tilde{\mathcal{A}}), \mathbf{w}) = \mathrm{val}(\gamma(\tilde{\mathcal{A}}), \mathbf{w}') = \mathrm{val}(\tilde{\mathcal{A}}, \mathbf{w}')$. $\square$

SUPPLEMENTARY REFERENCES

[1] Robert G Gallager, *Information theory and reliable communication*, Vol. 2 (Springer, 1968).
[2] RL Dobrushin, "General formulation of shannon's main theorem in information theory," Amer. Math. Soc. Trans **33**, 323–438 (1963).
[3] Sergio Verdu and Te Han, "A general formula for channel capacity," Information Theory, IEEE Transactions on **40**, 1147–1157 (1994).
[4] Wassily Hoeffding, "Probability inequalities for sums of bounded random variables," Journal of the American statistical association **58**, 13–30 (1963).
[5] Hugo Gimbert and Youssouf Oualhadj, *Automates probabilistes: problémes décidables et indécidables*, Tech. Rep. (RR-1464-09 LaBRI, 2009).
[6] Hugo Gimbert and Youssouf Oualhadj, "Probabilistic automata on finite words: Decidable and undecidable problems," in *Automata, Languages and Programming* (Springer, 2010) pp. 527–538.
[7] Mika Hirvensalo, "Improved undecidability results on the emptiness problem of probabilistic and quantum cut-point languages," in *SOFSEM 2007: Theory and Practice of Computer Science* (Springer, 2007) pp. 309–319.
[8] Graham James Oscar Jameson, *The prime number theorem*, Vol. 53 (Cambridge University Press, 2003).
[9] Seymour Ginsburg, *The Mathematical Theory of Context Free Languages.[Mit Fig.]* (McGraw-Hill Book Company, 1966).
[10] Azaria Paz, *Introduction to probabilistic automata* (Academic Press, Inc., Orlando, FL, USA, 1971).
[11] Anne Condon and Richard J Lipton, "On the complexity of space bounded interactive proofs," in *Foundations of Computer Science, 1989., 30th Annual Symposium on* (IEEE, 1989) pp. 462–467.
[12] Vincent D Blondel, Vincent Canterini, *et al.*, "Undecidable problems for probabilistic automata of fixed dimension," Theory of Computing systems **36**, 231–245 (2003).
